# Deep learning methods improve linear B-cell epitope prediction

**DOI:** 10.1186/s13040-020-00211-0

**Published:** 2020-04-17

**Authors:** Tao Liu, Kaiwen Shi, Wuju Li

**Affiliations:** Institute of Military Cognition and Brain Sciences, Academy of Military Medical Sciences, Taiping Road 27, Haidian district, Beijing, 100850 China

**Keywords:** Deep learning, Prediction, Linear B-cell epitope

## Abstract

**Background:**

B-cell epitopes play important roles in vaccine design, clinical diagnosis, and antibody production. Although some models have been developed to predict linear or conformational B-cell epitopes, their performance is still unsatisfactory. Hundreds of thousands of linear B-cell epitope data have accumulated in the Immune Epitope Database (IEDB). These data can be explored using the deep learning methods, in order to create better predictive models for linear B-cell epitopes.

**Results:**

After data cleaning, we obtained 240,563 peptide samples with experimental evidence from the IEDB database, including 25,884 linear B-cell epitopes and 214,679 non-epitopes. Based on the peptide center, we adapted each peptide to the same length by trimming or extending. A random portion of the data, with the same amount of epitopes and non-epitopes, were set aside as test dataset. Then a same number of epitopes and non-epitopes were randomly selected from the remaining data to build a classifier with the feedforward deep neural network. We built eleven classifiers to form an ensemble prediction model. The model will report a peptide as an epitope if it was classified as epitope by all eleven classifiers. Then we used the test data set to evaluate the performance of the model using the area value under the receiver operating characteristic (ROC) curve (AUC) as an indicator. We established 40 models to predict linear B-cell epitopes of length from 11 to 50 separately, and found that the AUC value increased with the length and tended to be stable when the length was 38. Repeated results showed that the models constructed by this method were robust. Tested on our and two public test datasets, our models outperformed current major models available.

**Conclusions:**

We applied the feedforward deep neural network to the large amount of linear B-cell epitope data with experimental evidence in the IEDB database, and constructed ensemble prediction models with better performance than the current major models available. We named the models as DLBEpitope and provided web services using the models at http://ccb1.bmi.ac.cn:81/dlbepitope/.

## Background

As B-cell epitopes play an important role in vaccine design, clinical diagnosis, and antibody production, a limited number of prediction models for linear or conformational B-cell epitopes have been developed [1, 2]. However, due to the easy operation of linear epitopes in experiments, it is highly desirable to develop accurate prediction models for linear B-cell epitopes, including traditional propensity scale-based methods [3–6] and machine learning-based models [7–18].

The traditional methods firstly assign some physicochemical properties to each amino acid (AA) in an antigen sequence. The commonly-used properties are hydrophilicity [3], flexibility [4], accessibility [5], and antigenicity [6]. In addition, there are 544 properties in the AAindex database [19]. Then a smoothing window was applied to calculate the average index along the antigen. Eventually, the peptides flanking the local maximum average index were taken as the potential B-cell epitopes. However, a study conducted by Blythe and Flower demonstrated that propensity scale-based methods are only marginally better than random ones [20].

To improve the performance, some machine learning-based models have been developed [7–18]. For example, based on the training dataset of 700 B-cell epitopes and 700 non-B-cell epitopes, the ABCpred model was developed using recurrent neural network [7]. The accuracy of five-cross-validation was 65.93%. In Bepipred1.0 [8], the combination of Hidden Markov Model and propensity scale methods was applied. In Bepipred2.0 [11], the random forest methods were applied to develop models with the annotated PDB data as the training and test datasets. Especially, the support vector machines were extensively used in developing models, which included AAP [12], BCpred and FBCpred [13], AAPpred [14], Bayesb [15], LEPS [16], SVMTriP [17], BEST [18], Lbtope [9], and APCpred [10]. Although these models have been developed, their performance is still away from satisfactory level. For example, the newly developed BepiPred2.0 [11] model and previous version Bepipred1.0 [8] were assessed on a test dataset, which contained 11,839 positive and 18,722 negative validated peptides obtained from the Immune Epitope Database (IEDB) [21]. The results indicated that the values of area under the receiver operating characteristic (ROC) curve (AUC) were only 57.40 and 54.80%, respectively. Therefore, it is necessary to develop more accurate models. In the present study, we explored the application of feedforward deep neural network for developing prediction models for linear B-cell epitopes.

## Methods

This work aimed to improve performance of linear B-cell epitope prediction by developing new models. To ensure the quality of training and testing samples, the sample data was obtained from the IEDB database and only those with experimental evidence were used. Feedforward deep neural network based classifiers were trained on these data and used as an ensemble model to improve prediction performance.

### IEDB dataset

To develop prediction models for linear B-cell epitopes, it is necessary to collect experimentally-confirmed datasets. To date, some databases for B-cell epitopes have been developed [1, 2]. However, the IEDB database is the most comprehensive database [21], containing the largest number of confirmed epitopes and non-epitopes. Therefore, we chose the IEDB database to develop the models.

For this purpose, we initially downloaded all 408,251 IEDB entries (March 28, 2018). Each entry contained 114 fields, such as host, antigen sequence name, peptide, and the start and end positions of peptide on the antigen sequence. From the 408,251 entries, we extracted 377,839 peptides as linear epitopes or non-epitopes. Through data washing strategies such as having full antigen sequence, unique epitope IRI accession number (IEDB ID), and peptide length located in the interval (10, 50], we finally achieved 240,563 peptides. Here, we considered the peptides with some properties, including “Positive”, “Positive-High”, “Positive-Intermediate”, or “Positive-Low” as the positive samples, which led to obtain 25,884 positive samples. The remaining were 214,679 negative samples. These IEDB peptides have different lengths as well.

To determine the optimal length for linear B-cell epitope prediction, we applied the traditional truncation and extension technique to generate a series of IEDBx datasets (x = 11, 12, …, 50), in which x represents the length of extracted peptides. We firstly downloaded 6086 antigen sequences from National Center for Biotechnology Information (NCBI) database using antigen sequence’s names associated with the samples. Then, we mapped each IEDB peptide on the antigen sequence. The integer part of the average of start and end positions was taken as the center position. According to the center position and the expected peptide length x, the corresponding peptides were extracted, and the IEDBx datasets were then constructed. Subsequently, duplicates were checked to ensure that all samples were unique in each dataset. An IEDBx dataset containing peptides of length x was used to build a model predicting epitopes of length x. The series of IEDBx datasets were used to build models predicting epitopes of length from 11 to 50, and thus determine the optimal length for epitope prediction.

### Lbtope_Fixed dataset

To compare performance between our models and existing models, it is necessary to construct a large independent test dataset. We downloaded the Lbtope_Fixed dataset from the Lbtope web server as one of the third-party datasets [9]. It was derived from peptides not less than 5 AAs in IEDB database, and published in 2013, containing 12,063 positive and 20,589 negative samples with 20 AAs long. We removed the intersection of Lbtope_Fixed and IEDB20 from Lbtope_Fixed. Therefore, the rest of the Lbtope_Fixed dataset had not been seen by our model and could be used fairly to compare our method with other methods. The rest of the Lbtope_Fixed dataset contained 8661 positive and 16,492 negative samples which can be downloaded from http://ccb1.bmi.ac.cn:81/dlbepitope/independenttest/Lbtope_Fixed.csv.

### ABCpred16 dataset

The ABCpred [7] web server provided an independent test dataset for comparing performance of different models. It contains 109 positive and 200 negative samples with 16 AAs long, and another 78 positive samples longer than 16 AAs, which generated 78 additional samples with 16 AAs long through the traditional truncation and extension technique. After carefully checking, three samples with unknown amino acid letter and two repeat samples were deleted. Also, we removed the intersection of ABCpred16 and IEDB16 from ABCpred16. Therefore, the rest of the ABCpred16 dataset had not been seen by our model and could be used fairly to compare our method with other methods. The rest of ABCpred16 dataset contained 107 positive and 196 negative samples which can be downloaded from http://ccb1.bmi.ac.cn:81/dlbepitope/independenttest/ABCpred16.csv.

### Feature extraction

There are 20 amino acids that make up peptides. Dipeptide means two amino acids joined by a single peptide bond. So, there are 400 possible dipeptides. For a peptide of length n, it can be divided into n-1 dipeptide. The fractions of all 400 dipeptides in a peptide form a vector of 400 elements named dipeptide composition, whose elements should add up to 1. Therefore, each peptide is represented by its dipeptide composition. We applied dipeptide composition to describe each peptide (epitope or non-epitope). And these dipeptide composition vectors were utilized for developing prediction models. In fact, dipeptide composition has been used to obtain amino acid pair antigenicity scale in AAP model [12].

### Ensemble deep learning methods

**Fig. 1:**
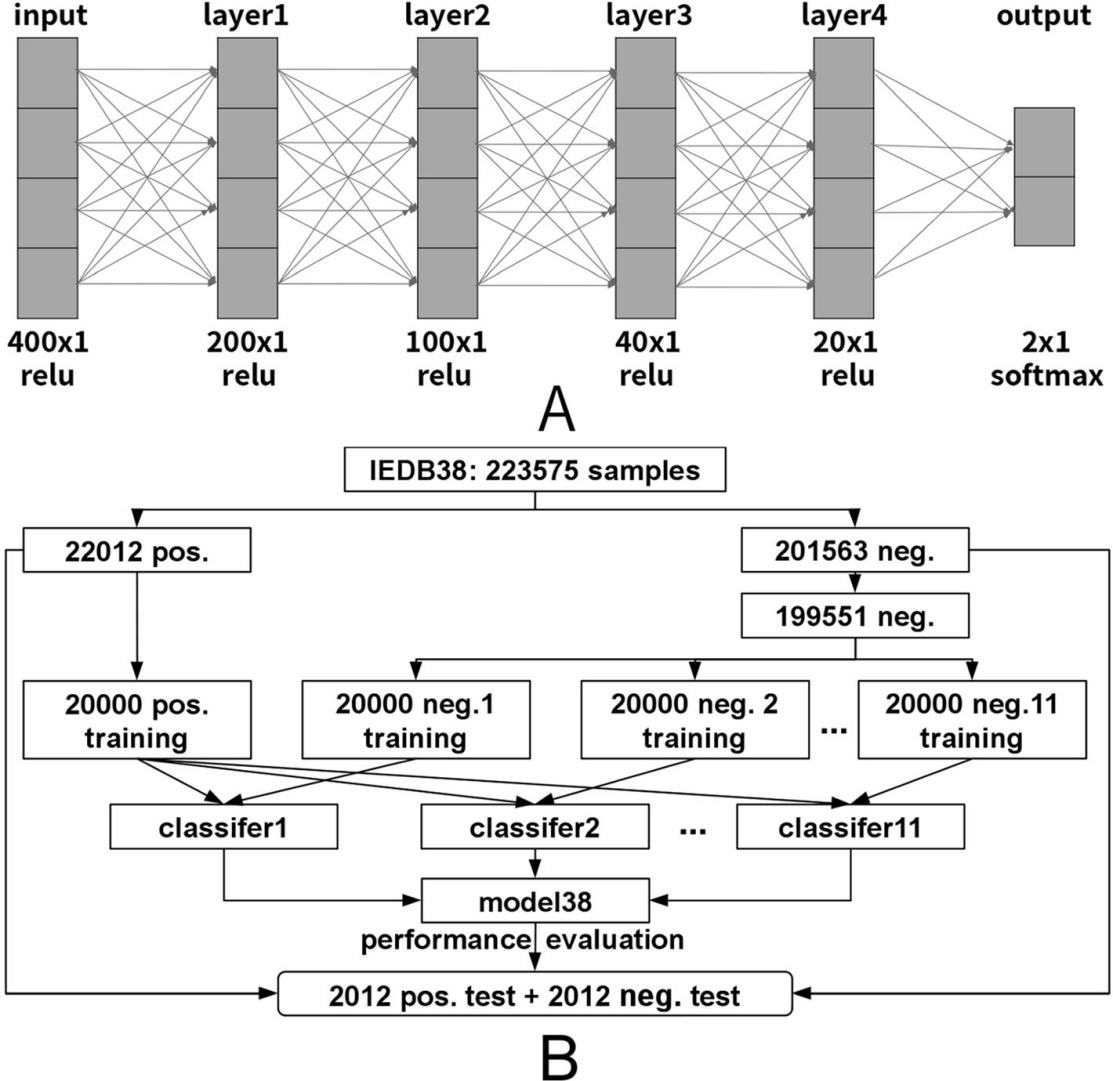
Architecture of feedforward deep neural network (panel **a**) and flowchart of ensemble model development using IEDB38 dataset as an example (panel **b**). “pos” and “neg” mean positive and negative samples; “test” stands for test dataset, and “training” for training dataset

In IEDBx datasets, the numbers of positive samples approximately ranged from 21 to 24 thousands, and the numbers of negative samples approximately ranged from 196 to 211 thousands. Therefore, ensemble learning was adopted to deal with the imbalance in the number of negative and positive samples. We used subsampling to make child datasets with diversity and balanced samples. For each IEDBx dataset, 20 thousands positive samples were randomly selected as the positive training set. The remaining positive samples were taken as the hold-out positive test set, and the same amount of negative samples were randomly selected as the hold-out negative test set. As a pool of negative training samples, the remaining negative samples were used to generate random subsample containing 20 thousands samples. The training set and hold-out test set can be obtained from http://ccb1.bmi.ac.cn:81/dlbepitope/index.php?r=/site/independenttest. The positive training set and a negative subsample made up a child training set with positive and negative samples balanced. Totally 11 negative subsamples were generated, and thus 11 child training sets were created, from which 11 classifiers were generated. These classifiers were combined as an ensemble model to score candidate epitope. If a peptide was predicted as epitope by a classifier, one was assigned; otherwise, zero was given. When all 11 classifiers were applied, 11 values achieved. The sum of these 11 values was taken as an index to indicate whether a peptide is an epitope. Obviously, if the sum is 11, the peptide will have the strongest signal to be an epitope. The procedure of ensemble learning is illustrated in Fig. 1b, in which IEDB38 dataset was used as an example to demonstrate the process. Finally, the AUC value from the hold-out test set was used to evaluate the performance of the ensemble model. And the allocation of training and hold-out test sets was selected to ensure the stable performance on hold-out test sets.

The classifiers were generated by the feedforward deep neural network. The R package “keras” was used to build the network. The input layer is a vector of 400 elements, which are dipeptide compositions from each peptide. The output layer contained 2 units corresponding to epitope and non-epitope respectively, which had a full connection with the previous layer. The activation function “softmax” was used for the output layer. Here, we set up “categorical_crossentropy” as the loss function, “optimizer_rmsprop” as the optimizer function, and “accuracy” as the metric. The network was evaluated on the corresponding hold-out test set. The R package “tfruns” was used to tune the hyperparameters for an evaluation accuracy as high as possible. To prevent overfitting, it’s usually best to start with relatively few layers and parameters, then begin increasing the size of the layers or adding new layers. We started with one hidden layer and ended up with four hidden layers with evaluation accuracy reaching about 0.8. The number of units in the hidden layers was 200, 100, 40, and 20, respectively. The detailed architecture is provided in Fig. 1a. Additionally, the rate 0.4 was assigned for each layer dropout to prevent overfitting, and the activation function “relu” was used as well. The other parameters are as follows: shuffle = TRUE, epochs = 400, batch_size = 8000, and validation_split = 0.2, in which the last parameter means that 20% of samples in the training dataset were used as the validation set to optimize the training parameters.

Additionally, because performance of models measured by accuracy, sensitivity, specificity, positive prediction rate, or Mathew’s correlation coefficient was dependent on the parameters used, herein, we applied AUC value, which was not dependent on the parameters used, to compare the performance of different models. Here, the R package “pROC” was used to calculate AUC value [22].

### Performance stability analysis

To determine whether the performance was associated with the sampling strategy to form the training and test datasets, we developed 10 models for IEDBx through repeatedly using the procedures as demonstrated in Fig. 1b by ten times, and correspondingly 10 AUC values were obtained from the test datasets. The standard deviation of the ten AUC values was used to evaluate the performance stability.

## Results

### Optimal length for prediction of linear B-cell epitope

To determine the optimal length for prediction of linear B-cell epitope, each IEDBx dataset was applied to extract the training and test datasets separately. Considering that the number of negative samples is far larger than that of positive samples, we adopted the ensemble learning and feedforward deep neural network to develop predictive models. The detailed processes for developing models are illustrated in Fig. 1b, in which IEDB38 was used as an example to demonstrate the process.

The IEDB38 dataset contains 22,012 positive and 201,563 negative samples. We randomly extracted 20,000 positive samples as the positive training set. The remaining 2012 positive samples were taken as the independent positive test set. Then, 2012 negative samples were randomly extracted as the independent negative test sets. The remaining 199,551 negative samples were applied to establish 11 negative training sets through sampling with replacement, in which each set contains 20,000 samples. The third step is to develop a prediction ensemble model, DLBEpitope, using the positive training set and each of 11 negative training set.

**Fig. 2:**
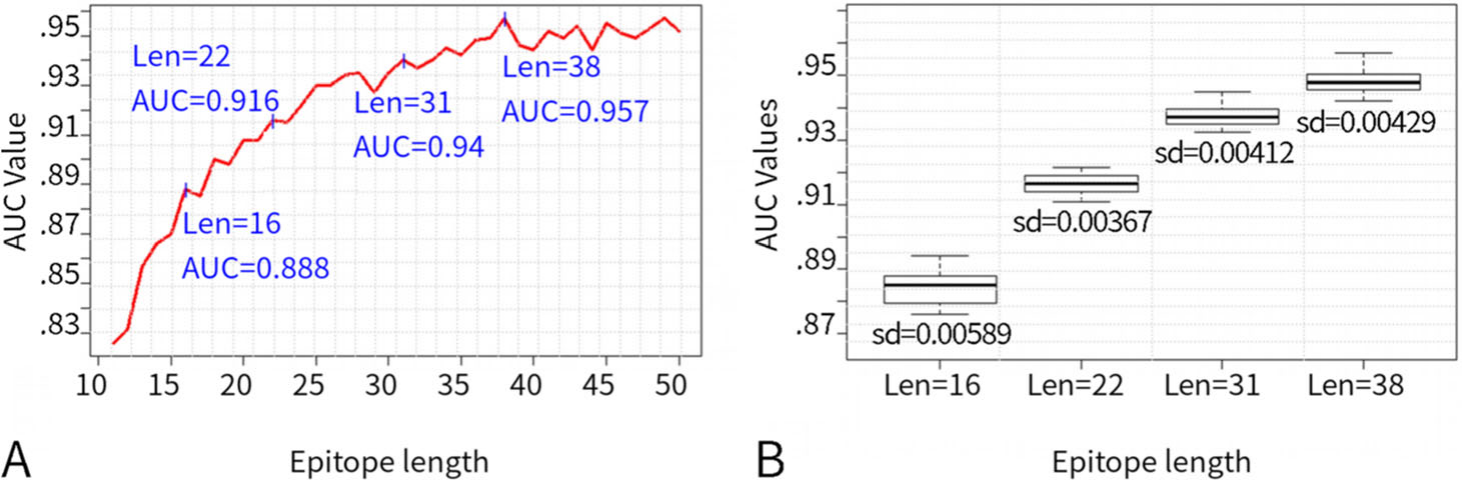
Models for different epitope length result in different AUC values (panel **a**), while the AUC values keep stable for models with the same epitope length (panel **b**). The AUC values were calculated using test datasets. The “sd” stands for standard deviation of 10 AUC values

For another IEDBx dataset, each training set was also composed of 20,000 positive and 20,000 negative samples. However, the number of test samples may be different. We used the AUC value from the independent test set to evaluate the performance of the ensemble model. The relationship between the epitope lengths and AUC values was displayed in Fig. 2a. It can be seen that the AUC values gradually increased with the epitope length. When the length reached 38, the AUC values become relatively stable. Herein, we selected values of epitope length equal to 16, 22, 31, and 38 for conducting further AUC stability analysis. The length of 16 was chosen because this length was often used in other models, such as ABCpred [7] and APCpred [10]. The other length values equal to 22, 31, and 38 were also selected because the corresponding AUC values of these lengths were local maximum, as shown in Fig. 2a.

### The DLBEpitope models are stable

We developed ten ensemble models for each IEDBx (x = 16, 22, 31, 38) through repeating ten times the procedure as demonstrated in Fig. 1b, and obtained ten AUC values. The boxplots of these AUC values are illustrated in Fig. 2b, in which it can be seen that the AUC values were maintained, which implied that the performance of the ensemble models is stable.

### Comparing with other models

We applied three datasets to compare the performance of DLBEpitope model with ABCpred [7], BepiPred1.0 [8], BepiPred2.0 [11], AAPpred [14], and APCpred [10] models. The first dataset was derived from each IEDBx (x = 16, 18, 20, 22, 31, and 38). Here, the lengths of 16, 18, and 20 were selected because ABCpred [7] and APCpred [10] models only provided these options. Additionally, the AAPpred [14] model only considers the length of 20 AAs. The reason to choose the lengths of 22, 31, and 38 is that the AUC values of these lengths were the local maximum (Fig. 2a). The second dataset was Lbtope_Fixed, which contained 8661 epitopes and 16,492 non-epitopes. Each sample had 20 AAs long. The third dataset was ABCpred16 with 107 positive and 196 negative samples. All three datasets can be downloaded from our web server as well.

### Utilizing the DLBEpitope dataset to compare the performance of different models

**Fig. 3:**
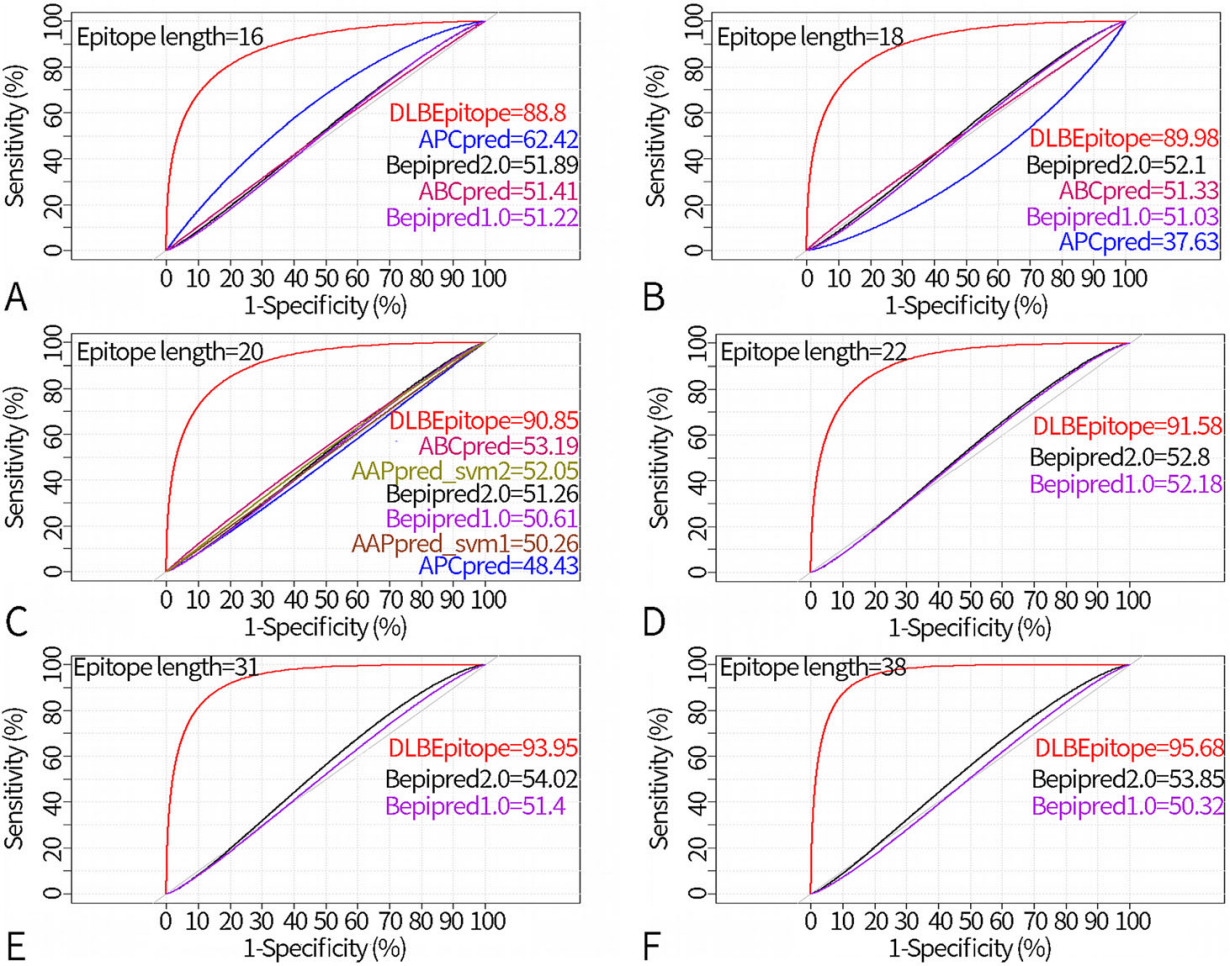
Performance comparisons among various models, including DLBEpitope, APCpred, Bepipred2.0, Bepipred1.0, ABCpred, AAPpred_svm1 and AAPpred_svm2 using DLBEpitopeX-test datasets (X is 16, 18, 20, 22, 31 and 38 for panels **a**-**f** separately). Epitope lengths of each dataset and AUC values for each model are displayed

Here the test datasets obtained from IEDBx (x = 16, 18, 20, 22, 31 and 38) were used to compare the performance of the ABCpred [7], APCpred [10], AAPpred [14], BepiPred1.0 [8], BepiPred2.0 [11] models and our model DLBEpitope. For each IEDBx, the test dataset had no overlap with the training dataset, which led to guarantee a fair comparison. The number of samples in the test datasets was 6922, 6614, 6454, 6072, 4990, and 4024, respectively, in which the number of positive samples was as same as the number of negative samples. In view that some models only predict certain length values of epitopes, the detailed strategies for making comparison are as follows: DLBEpitope16-test, DLBEpitope18-test, and DLBEpitope20-test for ABCpred [7], APCpred [10], BepiPred1.0 [8], BepiPred2.0 [11], and DLBEpitope models; DLBEpitope22-test, DLBEpitope31-test, and DLBEpitope38-test for BepiPred1.0 [8], BepiPred2.0 [11], and DLBEpitope models. Additionally, the AAPpred model [14] was incorporated into the DLBEpitope20-test dataset-based comparison. The ROC plots and the corresponding AUC values are illustrated in Fig. 3 and Table 1, in which two conclusions were drawn. The first conclusion is that the DLBEpitope model has the best AUC value for each epitope length. The second one is that the Bepipred2.0 model [11] has superior performance than that of Bepipred1.0 model [8], which is consistent with the results obtained from a previous study [11].

**Table 1:** Linear B-cell epitope prediction performance of various methods

Method	Performance (AUC values) on datasets							
	IEDB16	IEDB18	IEDB20	IEDB22	IEDB31	IEDB38	Lbtope_Fixed	ABCpred16
AAPpred_svm1	NA	NA	50.26	NA	NA	NA	53.01	NA
AAPpred_svm2	NA	NA	52.05	NA	NA	NA	53.83	NA
ABCpred	51.41	51.33	53.19	NA	NA	NA	51.54	55.29
APCpred	62.42	37.63	48.43	NA	NA	NA	51.01	61.19
Bepipred1.0	51.22	51.03	50.61	52.18	51.4	50.32	55.79	61.88
Bepipred2.0	51.89	52.10	51.26	52.8	54.02	53.85	53.23	55.33
DLBEpitope	88.80	89.98	90.85	91.58	93.95	95.68	73.83	65.5

*NA* the method does not predict epitope of this length

For ABCpred [7] and APCpred [10] models, all peptides from each test dataset were linked together and were submitted to the ABCpred web server or were predicted using in-house program, in which the options were set up to ensure that the potential epitopes were obtained as many as possible. Finally, all peptides and their scores in each test dataset were achieved through the intersection set of the prediction results and the relevant test datasets. Those scores were used to calculate AUC values.

For the Bepipred1.0 model [8], all peptides from each test dataset were submitted to the Bepipred1.0 web server with the default parameters. The outputs were the list of residues and their scores in each peptide. To obtain AUC values, each peptide was assigned a score by the average, minimum or maximum of the residue scores in the peptide, respectively. We found that the average-based AUC values were always the largest. Therefore, the average-based ROCs and associated AUC values were provided.

For the Bepipred2.0 model [11], we downloaded and ran the software locally. Then, the processes similar to those used for Bepipred1.0 [8] were used to calculate the average-based AUC values.

For the AAPpred model [14], we only considered the DLBEpitope20-test dataset, because the web server only predicted the peptides of 20 AAs long. Furthermore, the web server only predicted one peptide each time. Herein, we wrote a local R program AAPpred to submit the peptides to the web server and automatically fetch the prediction results. This model provided two prediction results for each sample, one from the combination of amino acid pair antigenicity (AAP) and amino acid propensity scales (AAPpred_svm1), the other from only the AAP (AAPpred_svm2). The AUC values were calculated using the prediction results as well.

For the DLBEpitope model, we firstly calculated the dipeptide compositions of each sample in each test dataset. Then, the DLBEpitopeX model, which was composed of 11 classifiers, was applied to predict each sample. In other words, each sample was assigned 1 (positive) or 0 (negative) by 11 times. The sum of these 11 values were used to calculate AUC values for the lengths of 16, 18, 20, 22, 31, and 38, respectively. Additionally, all samples were also directly submitted to the DLBEpitope web server. Then, each peptide was given a score ranged from 0 to 11 in order to calculate AUC values.

### Utilizing the Lbtope_Fixed and ABCpred16 datasets to compare the performance of different models

**Fig. 4:**
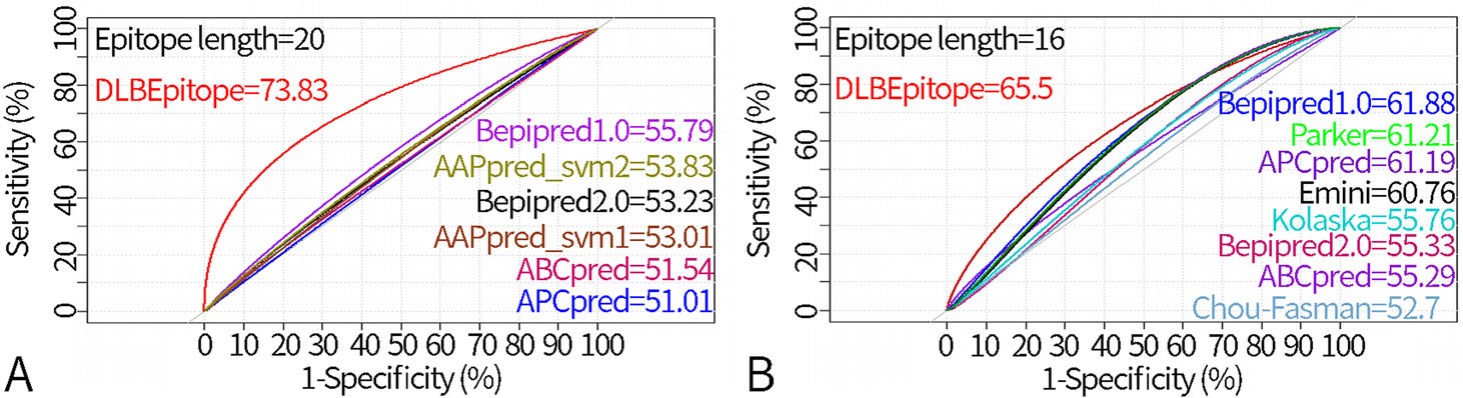
Performance comparisons among various models, including DLBEpitope, APCpred, Bepipred2.0, Bepipred1.0, ABCpred, AAPpred_svm1, AAPpred_svm2, Parker, Emini, Kolaska and Chou-Fasman using public test datasets Lbtope_Fixed (panel **a**) and ABCpred16 (panel **b**). Epitope lengths of each dataset and AUC values for each model are displayed

With the intersection of IEDB20 and Lbtope_Fixed removed, the modified Lbtope_Fixed test dataset contained 8661 positive and 16,492 negative samples. To the best of our knownledge, this is the largest third-party dataset for comparing the performance of different models. Therefore, the dataset facilitated making objective comparison on performance among the models of DLBEpitope, ABCpred [7], Beprpred1.0 [8], Bepipred2.0 [11], AAPpred_svm1.0, AAPpred_svm2 [14],and APCpred [10]. According to the prediction results, the AUC values were calculated and displayed in Fig. 4a and Table 1. It can be clearly seen that our model, DLBEpitope, has the best performance with the AUC value of 73.83%, which is far larger than 55.79%, as the second largest AUC value derived from the Bepipred1.0 model [8].

With the intersection of IEDB16 and ABCpred16 removed, the modified ABCpred16 dataset was used to compare the performance of the following models: ABCpred [7], Bepipred1.0 [8], Bepipred2.0 [11], APCpred [10], Parker Hydrophilicity-based Prediction (Parker) [3], Emini Surface Accessibility-based Prediction (Emini) [5], Kolaskar & Tongaonkar Antigenicity-based prediction (Kolaskar) [6], Chou & Fasman Beta-Turn-based Prediction (Chou-Fasman) [23], and our model (DLBEpitope16). The models of Chou_Fasman, Emini, Parker and Kolaskar were run on the IEDB web server [21]. According to the prediction results, the AUC values were calculated and illustrated in Fig. 4b and Table 1. Although our model performed best, it was not significant compared with other models. It might be due to the very small size of this dataset, containing only 107 positive and 196 negative samples. The limited number of samples couldn’t distinguish the performance of different models, thereby necessitating the presence of more samples.

### Developing DLBEpitope web server

According to the models developed here, we developed a web server to provide service for linear B-cell epitope prediction. Users can paste the antigen sequences in FastA format into the sequence window, or provide a file containing antigen sequences in FastA format. Then, the DLBEpitopeX model was chosen and a threshold (0 ~ 11) was adopted to predict linear B-cell epitopes. The prediction results can be fetched by providing task ID or email notification or refreshing the webpage. The detailed information can be found in http://ccb1.bmi.ac.cn:81/dlbepitope/.

## Discussion

In this study, we applied feedforward deep neural network to develop a prediction model for linear B-cell epitope with dipeptide compositions as features. Comparing to the existing machine learning-based models, including ABCpred [7], BepiPred1.0 [8], BepiPred2.0 [11], AAPpred [14], and APCpred [10], our model possessed four advantages. The first one was that the model DLBEpitopeX had the best performance on DLBEpitopeX-test dataset, as well as two public test datasets (Lbtope_Fixed and ABCpred16). The second advantage was that all samples in both training and test datasets were achieved from the experiments. However, the negative samples in models of ABCpred [7], Bepipred1.0 [8], AAPpred [14], and APCpred [10] were randomly generated from the SWISS-Prot database. The third one was that we had the largest number of samples in training datasets, which could better statistically describe features of linear B-cell epitopes. In fact, each classifier in DLBEpitopeX model was trained on a dataset of 20,000 positive and 20,000 negative samples, of which the number of samples was far greater than those of the existing models. For example, the Lbtope model [9] was trained on a dataset with about 10,000 positive and 18,500 negative samples, which involved the largest number of samples in the training dataset in the existing models. The fourth was that, to the best of our knowledge, the deep learning methods were used for the first time to develop prediction models for linear B-cell epitopes. Eventually, we developed a web server to provide better support for linear B-cell epitope prediction.

As shown in Fig. 3, our method performed far better than other methods on the test dataset derived from the 2018 IEDB database. These test datasets were randomly selected and not used for training our models. And overfitted models usually performed poorly in predicting test dataset they had not seen before. The performance improvement may be due to the large training dataset with experimental evidences and the combination of ensemble learning and deep neural network. The large training dataset provided more comprehensive recognition information. Multiple deep neural networks can learn more complex and accurate distinguishing information from multiple aspects. And ensemble learning can combine these networks to achieve much stronger generalization ability and make more accurate predictions.

However, on two existing public datasets, the Lbtope_Fixed and ABCpred16 datasets, our method performed much worse than on the IEDBx test datasets (Fig. 4). The Lbtope_Fixed dataset consisted of samples of 20 AAs in length and was derived from peptides not less than 5 residues in the 2012 edition of IEDB, whereas the IEDB20 was derived from epitopes with 10 to 50 residues in the 2018 edition of IEDB. Removing the overlap with IEDB20, the remained Lbtope_Fixed data should be derived only from epitopes of length 5 to 9 or greater than 50 in the 2012 edition of IEDB. This may lead to some bias, and thus make our method perform worse than when tested with IEDB20 test dataset. Even though, our method performed far better on the final Lbtope_Fixed dataset than other methods. The ABCpred16 dataset consisted of samples of 16 AAs in length. We have also removed the intersection of ABCpred16 and IEDB16 to make sure that the rest of the ABCpred16 has not been seen by our models. On the remaining ABCpred16 dataset, our method did not significantly outperformed the other methods. The reason may be that the remained ABCpred16 dataset is too small (107 positive and 196 negative samples), which likely lead to some bias.

Except for the features of dipeptide compositions, we also explored the application of AAindex [19] database in predicting linear B-cell epitopes. In AAindex database [19], each amino acid was represented by 544 physicochemical properties. Therefore, each peptide was described by the average of 544 indexes, among which there were 13 properties with missed values. Each peptide was finally represented by a vector with 531 elements. According to the IEDBx and DLBEpitopeX-test datasets (X = 16, 22, 31, and 38), the relevant models were developed and assessed. The AUC values for epitope lengths of 16, 22, 31, and 38 were 74.83, 77.54, 79.64, and 81.32%, respectively, which were less than the AUC values obtained from the dipeptide compositions-based model of DLBEpitopeX (X = 16, 22, 31, and 38). Therefore, the AAindex-based models were not used in our web server.

Finally, it should be mentioned that herein some machine learning-based models have not been used for comparing the performance because of the following reasons. The Bcpred [13] web server provides options for the models of BCpred, FBCpred, and AAP. However, the web servers cannot provide complete prediction results for the test datasets. For example, for the ABCpred16 dataset containing 107 positive and 196 negative samples, there were 29 positive samples overlapping with the positive training dataset obtained from the BCpred model. The remaining 78 positive and 196 negative samples were submitted to the web server by setting up the specificity of 65% to ensure that more prediction results were available. The prediction only returned the scores for 136 samples. There were 138 samples without scores. Therefore, the ROC plots and related AUC values couldn’t be provided. Additionally, the speed of running for SVMTrip [17] and Lbtope [9] web servers was very slow. Finally, we couldn’t access Besysb [15], LEPs [16],and BEST [18] web servers. In summary, these eight models were not used for comparing the performance.

In future, we will attempt to incorporate more samples into the development of prediction models for linear B-cell epitopes. In fact, the population space for linear B-cell epitopes was very large. For example, the population for epitope length of 16 contained 20^16^ (6.5536e+20) possible samples, which was far larger than 233,362, the number of samples in IEDB16 dataset. The ratio of 233,362 and 6.5536e+20 is only 3.560822e-16. Therefore, more samples are required to develop accurate prediction models for linear B-cell epitopes. Additionally, using more and more samples, deep learning methods will play a key role in developing prediction models not only for B-cell epitopes, but also for other biological problems.

## Conclusions

Trained on the large amount of linear B-cell epitope data with experimental evidence in the IEDB database, ensemble deep learning improved the performance of linear B-cell epitope prediction.

## Supplementary information

**Additional file 1.**

## Data Availability

The data that support the findings of this study are available from http://ccb1.bmi.ac.cn:81/dlbepitope/. The detailed web link is included in this article.

## Abbreviations

AA: Amino acid
AAP: Amino acid pair;
AUC: Area under the receiver operating characteristic curve
IEDB: Immune Epitope Database
NCBI: National Center for Biotechnology Information
ROC: Receiver operating characteristic;

## References

[1] Dhanda SK, Usmani SS, Agrawal P, Nagpal G, Gautam A, Raghava GPS (2017). Novel in silico tools for designing peptide-based subunit vaccines and immunotherapeutics. Brief Bioinform.

[2] Potocnakova L, Bhide M, Pulzova LB (2016). An introduction to B-cell epitope mapping and in Silico epitope prediction. J Immunol Res.

[3] Parker JM, Guo D, Hodges RS (1986). New hydrophilicity scale derived from high-performance liquid chromatography peptide retention data: correlation of predicted surface residues with antigenicity and X-ray-derived accessible sites. Biochemistry..

[4] Karplus PA, Schulz GE (1985). Prediction of chain flexibility in proteins. Naturwissenschaften..

[5] Emini EA, Hughes JV, Perlow DS, Boger J (1985). Induction of hepatitis a virus-neutralizing antibody by a virus-specific synthetic peptide. J. Virol..

[6] Kolaskar AS, Tongaonkar PC (1990). A semi-empirical method for prediction of antigenic determinants on protein antigens. FEBS Lett.

[7] Saha S, Raghava GPS (2006). Prediction of continuous B-cell epitopes in an antigen using recurrent neural network. Proteins Struct Funct Bioinforma.

[8] Larsen JEP, Lund O, Nielsen M (2006). Improved method for predicting linear B-cell epitopes. Immunome Res.

[9] Singh H, Ansari HR, Raghava GPS (2013). Improved method for linear B-cell epitope prediction using Antigen’s primary sequence. PLoS One.

[10] Shen W, Cao Y, Cha L, Zhang X, Ying X, Zhang W (2015). Predicting linear B-cell epitopes using amino acid anchoring pair composition. BioData Min.

[11] Jespersen MC, Peters B, Nielsen M, Marcatili P (2017). BepiPred-2.0: improving sequence-based B-cell epitope prediction using conformational epitopes. Nucleic Acids Res.

[12] Chen J, Liu H, Yang J, Chou K-C (2007). Prediction of linear B-cell epitopes using amino acid pair antigenicity scale. Amino Acids.

[13] EL-Manzalawy Y, Dobbs D, Honavar V (2008). Predicting linear B-cell epitopes using string kernels. J Mol Recognit.

[14] Davydov II, Tonevitskiĭ AG (2009). Linear B-cell epitope prediction. Mol. Biol. (Mosk).

[15] Wee LJK, Simarmata D, Kam Y-W, Ng LFP, Tong JC (2010). SVM-based prediction of linear B-cell epitopes using Bayes feature extraction. BMC Genomics.

[16] Wang H-W, Lin Y-C, Pai T-W, Chang H-T (2011). Prediction of B-cell linear epitopes with a combination of support vector machine classification and amino acid propensity identification. J Biomed Biotechnol.

[17] Yao B, Zhang L, Liang S, Zhang C (2012). SVMTriP: a method to predict antigenic epitopes using support vector machine to integrate tri-peptide similarity and propensity. PLoS One.

[18] Gao J, Faraggi E, Zhou Y, Ruan J, Kurgan L (2012). BEST: improved prediction of B-cell epitopes from antigen sequences. PLoS One.

[19] Kawashima S, Kanehisa M (2000). AAindex: Amino Acid index database. Nucleic Acids Res.

[20] Blythe MJ, Flower DR (2009). Benchmarking B cell epitope prediction: underperformance of existing methods. Protein Sci.

[21] Vita R, Overton JA, Greenbaum JA, Ponomarenko J, Clark JD, Cantrell JR (2015). The immune epitope database (IEDB) 3.0. Nucleic Acids Res.

[22] Robin X, Turck N, Hainard A, Tiberti N, Lisacek F, Sanchez J-C (2011). pROC: an open-source package for R and S+ to analyze and compare ROC curves. BMC Bioinformatics.

[23] Chou PY, Fasman GD. Prediction of the secondary structure of proteins from their amino acid sequence. Adv Enzymol Relat Areas Mol Biol.1978;47:45–148. 10.1002/9780470122921.ch2 364941

